# Supplementary material for: The Winged Helix Domain of CSB Regulates RNAPII Occupancy at Promoter Proximal Pause Sites
Source: Int J Mol Sci. 2021 Mar 25;22(7):3379. doi: 10.3390/ijms22073379 (PMC8037043; doi:10.3390/ijms22073379)
Supplement: Supplementary file 1 [file ijms-22-03379-s001.pdf]

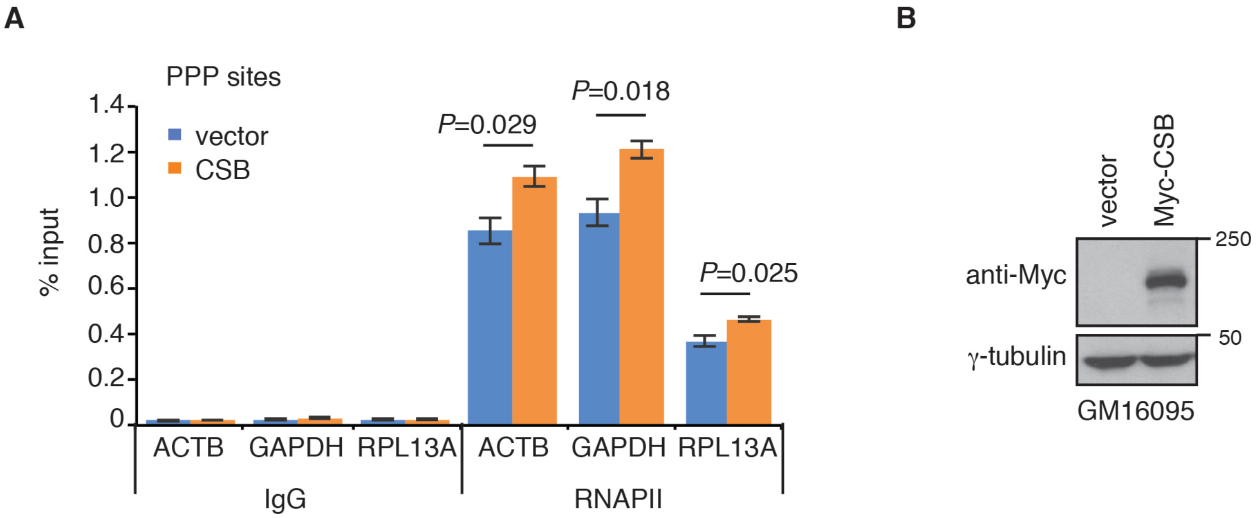

Supplementary Figure S1. Overexpression of Myc-CSB enhances RNAPII occupancy at PPP sites of *ACTB*, *GAPDH* and *RPL13A* genes in GM16095 cells. (**A**) RNAPII ChIP analyses of *ACTB*, *GAPDH* and *RPL13A* genes in GM16095 cells overexpressing the vector alone or Myc-CSB. Standard errors from three independent experiments are shown. *P* values were derived using a two-tailed Student's *t*-test. (**B**) Western analysis of GM16095 cells overexpressing the vector alone or Myc-CSB. Immunoblotting was performed with anti-Myc and anti-γ-tubuliln antibodies.

Supplementary Figure S2
Uncropped western images --- corresponding Figures are indicated

Figure 1A

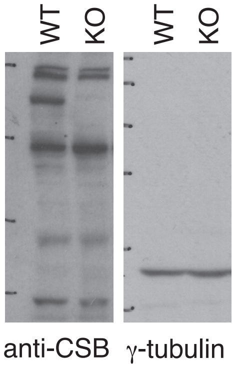

Figure 2A

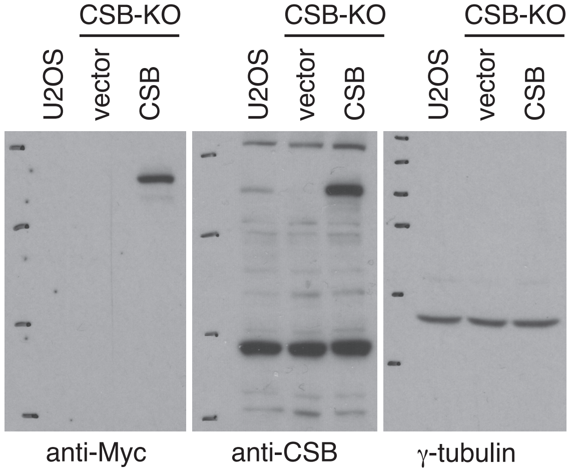

Figure 3A

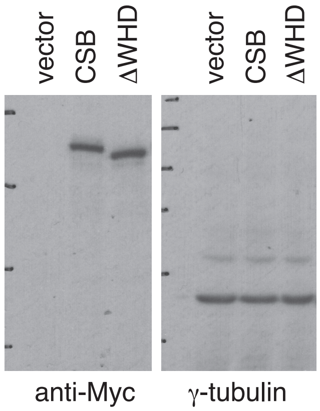

Figure 3D

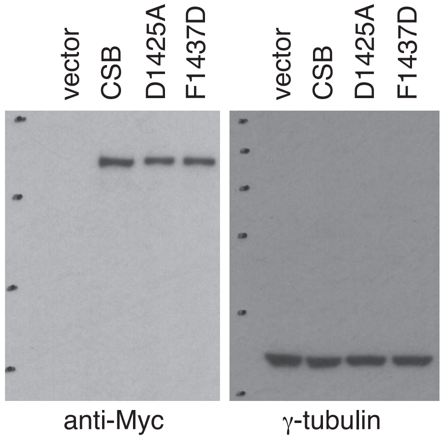

Figure 4C

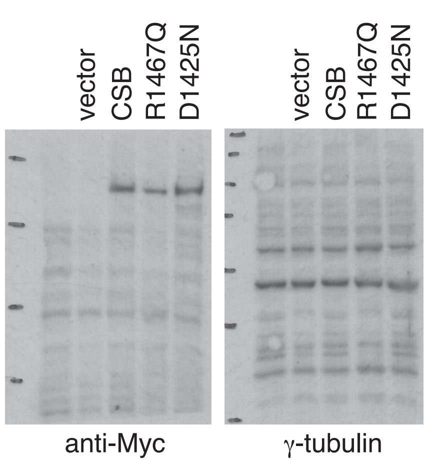

Figure 4D

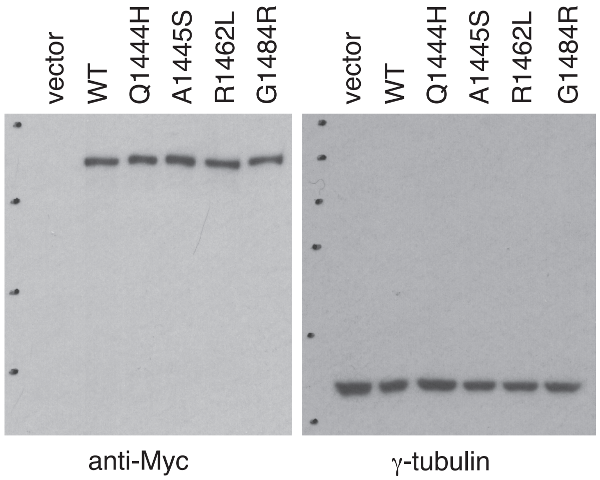

Supplementary Table S1

Q-PCR Primers [1]

Target	Forward primer	Reverse primer
ACTB_PPP	GAGGGGAGAGGGGGTAAA	AGCCATAAAAGGCAACTTTCG
ACTB_In5	GGAGCTGTCACATCCAGGGTC	TGCTGATCCACATCTGCTGG
ACTB_TES	GGGACTATTTGGGGGTGTCT	TCCCATAGGTGAAGGCAAAG
GAPDH_PPP	CTCCTGTTCGACAGTCAGC	TTCAGGCCGTCCCTAGC
GAPDH_In5	ATAGGCGAGATCCCTCCAA	TGAAGACGCCAGTGGAC
GAPDH_TES	CCCTGTGCTCAACCAGT	CTCACCTTGACACAAGCC
TEFM_PPP	CTTGGAGATGAGCGGGTCTG	GACAGACGGGAAATCACCCC
TEFM_In2	TGGCCAATGTGGTGAAAGCC	GGGACTACAGGCCCACGCC
TEFM_TES	ACCACATAGACTTTATGACAGAGAA	TCAATCCATGCTTGTGAAGCAAA
CALM3_PPP	TGCGGGCAGTGAGTGTGGAGG	ACGGGGATCAAGGTTCCTCCGG
CALM3_In1	GTTCGGGCCCCTATTGCGCAC	AAAAGCTGGCTCATTCGAGGCACC
CALM3_TES	GCGATGCCCGTTCTCTTGATC	CGCAGGGGAGTGTTGAAGAGAGA
MRPL21_PPP	CGTTACGCACGCGGTTC	GACCGTCAGGGAAGATGCTG
MRPL21_In4	CGGCATTGAGAATGGTTGCC	GCAATGATTGAGGCTCTCCT
MRPL21_TES	ACCAGGTTTCTGTGTTCTGGT	GGCCTGGTGCTTACAGACAT
RPL13A_PPP	ATGGCGGAGGTGCAGGTATG	AGAGAGGGTGCGACCCCATT
RPL13A_In6	AGATTTCAGGCCTGCTGAGG	CCGCAGACCATCGTGAGATA
RPL13A_TES	TGGCGTCTTTGCACTGTGTC	CTACCCTCTTCAAGCTCCTCAC
VCL_PPP	GTGAGGCTGGTTACGCCGAG	CGGGAACCGGCGAAGAGA
VCL_In6	CTCTGGTATCTGAATCTGCTTTCT	AGCAGTATTTGCAATGTTTGGTTT
VCL_TES	AGAACTTTTAGGTCAGGTTTCTCCT	GTTTCCAGATCTTGAGGATTAGTTT

[1] M.K.K. Shivji, X. Renaudin, C.H. Williams, A.R. Venkitaraman, BRCA2 Regulates Transcription Elongation by RNA Polymerase II to Prevent R-Loop Accumulation, Cell Rep, 22 (2018) 1031-1039.

Supplementary Table S2

Primers used to generate CSB mutations via site-directed mutagenesis

**CSB mutations**	
D1425A	CTGCTGCCCACCACAGAGCATGCTGACCTTCTGGTGGAG
D1425N	CCCACCACAGAACACAATGACCTACTAGTGGAGATGAGAAACTTC
F1437D	GGAGATGAGAAACTTCATCGCTGATCAGGCCCACACTGATGGC
Q1444H	GGCCCACACTGATGGCCACGCTAGCACCAGGGAGATAC
A1445S	CACACTGATGGCCAGTCCAGTACTAGGGAGATACTGCAG
R1462L	GAATCCAAGTTATCTGCTAGCCTGTCTTGTGTCTTCCGAGAACTATTG
R1467Q	CACAGTCTTGTGTCTTCCAAGAGCTCTTGAGAAATCTGTGCAC
G1484R	GAACTTCTGGTGGTGAAAGAATTTGGAAACTCAAGCCAG
